# Diagnostic accuracy and comparison of BIPSS in response to lysine vasopressin and hCRH

**DOI:** 10.1530/EC-18-0046

**Published:** 2018-02-12

**Authors:** Kush Dev Singh Jarial, Anil Bhansali, Vivek Gupta, Paramjeet Singh, Kanchan K Mukherjee, Akhilesh Sharma, Rakesh K Vashishtha, Suja P Sukumar, Naresh Sachdeva, Rama Walia

**Affiliations:** 1 Department of EndocrinologyPost Graduate Institute of Medical Education and Research, Chandigarh, India; 2 Department of Radio-diagnosisPost Graduate Institute of Medical Education and Research, Chandigarh, India; 3 Department of NeurosurgeryPost Graduate Institute of Medical Education and Research, Chandigarh, India; 4 Department of PsychiatryPost Graduate Institute of Medical Education and Research, Chandigarh, India; 5 Department of HistopathologyPost Graduate Institute of Medical Education and Research, Chandigarh, India

**Keywords:** bilateral inferior petrosal sinus sampling (BIPSS), hCRH (human corticotrophin releasing hormone), LVP (lysine vasopressin), contrast-enhanced magnetic resonance imaging (CEMRI)

## Abstract

**Context:**

Bilateral inferior petrosal sinus sampling (BIPSS) using hCRH is currently considered the ‘gold standard’ test for the differential diagnosis of ACTH-dependent Cushing’s syndrome (CS). Vasopressin is more potent than CRH to stimulate ACTH secretion as shown in animal studies; however, no comparative data of its use are available during BIPSS.

**Objective:**

To study the diagnostic accuracy and comparison of hCRH and lysine vasopressin (LVP) stimulation during BIPSS.

**Patients and methods:**

29 patients (27-Cushing’s disease, 2-ectopic CS; confirmed on histopathology) underwent BIPSS and were included for the study. Patients were randomized to receive hCRH, 5 U LVP or 10 U LVP during BIPSS for ACTH stimulation. BIPSS and contrast-enhanced magnetic resonance imaging (CEMRI) were compared with intra-operative findings of trans-sphenoidal surgery (TSS) for localization and lateralization of the ACTH source.

**Results:**

BIPSS correctly localized the source of ACTH excess in 29/29 of the patients with accuracy of 26/26 patients, using any of the agent, whereas sensitivity and PPV for lateralization with hCRH, 5 U LVP and 10 U LVP was seen in 10/10, 6/10; 10/10,8/10 and 7/7,6/7 patients respectively. Concordance of BIPSS with TSS was seen in 20/27, CEMRI with BIPSS in 16/24 and CEMRI with TSS in 18/24 of patients for lateralizing the adenoma. Most of the side effects were transient and were comparable in all the three groups.

**Conclusion:**

BIPSS using either hCRH or LVP (5 U or 10 U) confirmed the source of ACTH excess in all the patients, while 10 U LVP correctly lateralized the pituitary adenoma in three fourth of the patients.

## Introduction

The differential diagnosis of ACTH-dependent Cushing’s syndrome (CS) as pituitary or ectopic source of ACTH production is often challenging for the physicians as none of the biochemical tests or imaging modalities have 100% diagnostic accuracy (1). Cushing’s disease (CD) accounts for about 90–95% of the cases when there is no obvious source of ACTH hypersecretion, and this distinction is essential to decide further therapeutic strategies (2, 3, 4, 5). Sellar imaging fails to detect small adenomas in 50% of the patients with Cushing’s disease and further this is complicated by the fact that pituitary incidentaloma can be found in 10–30% of the general population (6). BIPPS demonstrated 100% sensitivity and specificity in differentiating pituitary from peripheral source of ACTH production in the earlier studies; however, recent studies have shown lower accuracy due to the false-positive (pseudo Cushing’s states and on medical treatment) and false-negative results (cyclical CS) (7, 8, 9). Ovine or hCRH and/or desmopressin have been used to stimulate corticotropes during BIPSS to improve the accuracy of the procedure for localizing the source of ACTH hypersecretion and were found to be equipotent in stimulating ACTH production (10, 11, 12). Animal studies have shown that vasopressin is more potent stimulus for ACTH secretion than CRH. However, human studies have shown that vasopressin is equipotent to hCRH for ACTH release (13, 14, 15). Desmopressin has higher affinity for V2 receptors present on the kidney as compared to that for V3 receptors present on pituitary. Further, vasopressin is more potent than desmopressin in ACTH release due to its higher affinity for V3 receptors present on pituitary, and it also increases the CRH release (16, 17). Vasopressin was used during BIPSS in a previous study; however, the patients were few (18).

The present study was performed to compare the diagnostic accuracy of human hCRH and lysine vasopressin for stimulating ACTH release during BIPSS in localizing and lateralizing the source of ACTH hypersecretion.

## Patients and methods

### Bilateral inferior petrosal sinus sampling (BIPSS)

The diagnosis of CS was based on clinical symptoms and signs, ACTH-dependent hypercortisolemia, non-suppressible cortisol dynamics, sellar imaging either normal or showing pituitary microadenoma and adenoma or carcinoid on histopathology following trans-sphenoidal or appropriate surgery. Twenty-nine consecutive patients of CS; twenty-seven patients of Cushing’s disease having microadenoma (confirmed on histopathology) and 2 patients of ectopic CS (bronchial carcinoid −1, thymic carcinoid 1) in whom BIPSS was performed were included in the study from April 2013 to November 2015. BIPSS was performed in the neuroradiology unit of the institute. All the patients included were hypercortisolic (raised midnight plasma cortisol, urinary free cortisol and non-suppressible cortisol on ONDST (overnight dexamethasone suppression test) or LDDST (low dose dexamethasone suppression test)) and had raised ACTH. ONDST was performed by orally administering a 1-mg tablet of dexamethasone at 23:00 h, and a blood sample for cortisol was collected at 08:00 h the next day. Plasma cortisol above 50 nmol/L was taken as non-suppressible. LDDST was performed by orally administering 0.5-mg tablets of dexamethasone at 09:00, 15:00, 21:00 and 03:00 h for 48 h, and blood samples for cortisol were collected at 08:00 h. Plasma cortisol above 50 nmol/L was taken as non-suppressible. On the day of BIPSS procedure, 08:00 h plasma cortisol was elevated in all the patients, thus excluding cyclicity. Bilateral inferior petrosal sinuses were catheterized using femoral route under local anesthesia. 5 French catheters were used to reach the bilateral petrosal sinuses. Once catheters were placed in the petrosal sinuses, position was confirmed using fluoroscopic guidance. Catheterization of the bilateral inferior petrosal sinuses was successful in all the patients. Simple random sampling using random number table was done for the selection of agent for stimulation during BIPSS. hCRH (CRH Ferring 100 µg, Unipharma SA) was used in 10 patients. 10 U LVP was used in 9 patients while 5 U LVP was used in 10 patients to find out the minimum effective dose of LVP for ACTH stimulation. LVP diluted in 10 mL of normal saline was given intravenously through peripheral catheter over six minutes, whereas 100 µg hCRH was given intravenously as a bolus through peripheral catheter after mixing it with the diluent. Blood samples were drawn at basal (−6 min), 0, 2, 3, 5, 10 and 15 min and were stored in pre-chilled EDTA tubes. The patients were monitored for blood pressure, heart rate, oxygen saturation or any other complaint reported by them. Samples were immediately transported to the endocrinology laboratory of the institute for processing. Standardized cut-off value for inferior petrosal sinus to peripheral venous ACTH of ≥2:1 for the basal and ≥3:1 after stimulation were considered to denote the pituitary source of ACTH excess, while inter-sinus ACTH ratio of ≥1.4:1 was taken for lateralization of ACTH source.

Ethical approval was taken from the Ethics Committee of PGIMER, Chandigarh, India. Informed written consent was obtained from all the patients.

### Imaging, trans-sphenoidal surgery and histopathology

Dynamic contrast enhanced magnetic resonance imaging (CEMRI) was done in all the patients with ACTH-dependent CS. Contrast-enhanced computerized tomography (CECT) chest and abdomen were done in patients with ectopic CS. Patients with Cushing’s disease underwent TSS, while resection of the respective tumor was performed in patients with ectopic CS. Intra-operative findings regarding tumor location, its tumor epicenter, tumor extension and so forth were recorded by surgeon during TSS and correlated with MRI and BIPSS. Histopathological findings were considered as the ‘gold standard’ for the diagnosis of CS.

### Assay

Serum cortisol and ACTH were measured by electro-chemiluminescence-immuno-assay (ECLIA) (ELECSYS-2010, Roche Diagnostics). Inter-and intra- assay CV was 2.3%, 6.4% and 1.4%, 2.8% for ACTH and plasma cortisol, respectively.

### Statistical analysis

SPSS, version 17 has been used for statistical analysis. Descriptive statistics has been used and expressed in the form of frequencies and proportions. Significance value has been considered at *P* ≤ 0.05. For calculating the sensitivity and specificity of various diagnostic tests, a gold standard for establishing diagnosis has been considered in the form of intra-operative localization and lateralization of tumour. Sensitivity and positive predictive derived for the various tests in this study by generating a classical 2 × 2 contingency cross table. Comparison of predictive values for the two stimulating agents was done using ‘N−1’ Chi squared test. For calculating the concordance for localizing and lateralizing the ACTH source, simple manual case-by-case comparison was done.

## Results

### Comparison and diagnostic accuracy of BIPSS for localizing and lateralizing ACTH source with hCRH or LVP

At the basal point of time (prior to stimulation), sensitivity and PPV of BIPSS for the localization of ACTH source was seen in 26/29 and 26/26 of patients, respectively. Further, BIPSS was able to localize the source of ACTH in 29/29 patients at 2 and 3 min after stimulation using LVP (either 5 U or 10 U) and hCRH, respectively.

At basal point of time (prior to stimulation), sensitivity for lateralization of pituitary adenoma during BIPSS was 22/27; and 16/22 of patients had accurate lateralization of the ACTH source. Post-stimulation during BIPSS, 27/27 patients lateralized pituitary adenoma with any of the stimulating agent, while PPV for lateralization of 6/10, 8/10 and 6/7 was observed with hCRH, 5 U LVP and 10 U LVP respectively.

Pituitary lesion ≤6 mm has a higher chance than lesion larger than 6 mm of being pituitary incidentaloma and being the real cause for hypercortisolism in the patient. The role of BIPSS becomes more important in this scenario. We tried to find out the performance of BIPSS in microadenoma ≤6 mm. In patients with pituitary adenoma ≤6 mm, BIPSS lateralized adenoma in 16/16 of the patients, while accuracy of lateralization was 3/4, 5/7 and 4/5 with hCRH, 5 U LVP and 10 U LVP respectively; which was further substantiated by histopathology. Lateralization with BIPSS correlated with the intraoperative findings during TSS in all the three patients who had normal CEMRI sella.

Maximum stimulated ACTH level of 20,000 pg/mL was achieved with 5 U LVP, while maximum ACTH level of 3848 pg/mL was obtained after hCRH stimulation (Table 1). Both the patients of ectopic CS did not achieve the basal and stimulated recommended cut-off of 2:1 and 3:1, respectively.

**Table 1:** Comparison of LVP 10 U, LVP 5 U and hCRH.

	Max ACTH (pg/mL)	Max ACTH ratio (C:P)	Max inter-sinus ratio	Sensitivity of lateralization post stimulation	Lateralization accuracy	Localization accuracy
AVP (10 U)	1687 (2 min)	140.1 (2 min)	27.5 (2 min)	9/9	6/7	9/9
AVP (5 U)	20000 (1 min)	122.8 (2 min)	57.6 (3 min)	10/10	8/10	10/10
hCRH	3848 (3 min)	146.8 (3 min)	59.3 (3 min)	10/10	6/10	10/10

TSS was performed in 27 patients. Patients with ectopic CS underwent surgery confirming bronchial carcinoid and thymic carcinoid in one patient each, respectively. 24 patients had microadenoma on CEMRI, while in three patients MRI was normal. Concordance of BIPSS and CEMRI in lateralizing adenoma was seen in 16/24 of the patients (4/8, 6/9 and 6/7 with hCRH, 5 U LVP and 10 U LVP respectively). Concordance in lateralizing adenoma between BIPSS and TSS was observed in 20/27 of patients (6/10, 7/9 and 7/8) with hCRH, 5 U LVP and 10 U LVP, respectively) and concordance of CEMRI with TSS was observed in 18/24 of patients.

Sixteen patients had pituitary adenoma size ≤6 mm. In these patients, concordance of BIPSS and CEMRI was seen in 12/16 of the patients (3/4, 5/7 and 4/5 with hCRH, 5 U LVP and 10 U LVP respectively), BIPSS with TSS in 13/16 patients (3/4, 5/7 and 5/5 with hCRH, 5 U LVP and 10 U LVP respectively), while for CEMRI and TSS concordance was observed in 12/16.

In seven patients in whom CEMRI was discordant with BIPSS, five patients had concordance of CEMRI with intra-operative localization during trans-sphenoidal surgery, suggesting that when CEMRI and BIPSS are discordant, then CEMRI has better correlation for the lateralization of the tumor during TSS. Table 2 shows details of the BIPSS, CEMRI and histopathology findings.

**Table 2:** Concordance between the various modalities for lateralization-CEMRI, BIPSS and TSS.

S. no	Age (Year)	Sex	Bilateral inferior petrosal sinus sampling				CEMRI size (mm)	CEMRI localization	TSS localization
			hCRH/LVP	Localization	Max.C:P ratio	Max.Inter-sinus ratio			
1	19	F	LVP 10 U	Right Pit	72.0	27.5	8 × 5	Right	Right
2	17	M	LVP 10 U	Right/Pit	26.6	2.8	4 × 3 × 1.6	Right	Right
3	25	F	LVP 10 U	Right/Pit	140.1	1.5	5 × 4 × 4	Left	Left
4	26	F	LVP 10 U	Right/Pit	29.2	15.7	6.6 × 5.9 × 4.5	Right	Right
5	46	F	LVP 10 U	Right Pit	39.2	27.3	4.8 × 4.3	Right	Right
6	20	M	LVP 10 U	Left/Pit	46.6	22.1	6 × 5	Left	Left
7	42	F	LVP 10 U	Left/Pit	3.39	2.39	6 × 4	Left	Left
8	35	M	LVP 10 U	Periphery	1.8	1.3	Normal	Normal	Ectopic
9	52	F	LVP 10 U	Periphery	0.99	1.22	Normal	Normal	Ectopic
10	23	F	LVP 5 U	Right/Pit	60.9	17.9	8.8 × 6.4 × 6.6	Right	Right
11	37	F	LVP 5 U	Left/Pit	59.8	33.2	5.3 × 4.5	Central	Left
12	42	F	LVP 5 U	Right/Pit	3.3	2.3	7.5 × 4.3 × 5	Left	Right
13	14	M	LVP 5 U	Right/Pit	11.5	9.2	4 × 5	Right	Right
14	23	F	LVP 5 U	Left/Pit	122.8	19.4	3 × 4	Left	Right
15	54	F	LVP 5 U	Right/Pit	4.6	3.5	5.1 × 4.5 × 4	Right	Right
16	35	F	LVP 5 U	Left/Pit	60.6	25.2	4.5 × 4.2	Left	Left
17	35	M	LVP 5 U	Right/Pit	23.4	57.6	5 × 4	Left	Left
18	40	M	LVP 5 U	Right/Pit	60.1	25.3	Normal	Normal	Right
19	32	F	LVP 5 U	Right/Pit	9.8	3.76	5.4 × 5.8	Right	Right
20	35	F	hCRH	Left/Pit	23.8	14.1	Normal	Normal	Left
21	18	F	hCRH	Right/Pit	47.3	41.3	5.7 × 3.8	Bilateral	Right
22	26	F	hCRH	Right/Pit	16.6	1.6	4 × 3	Right	Right
23	16	F	hCRH	Right/Pit	54.3	59.3	4.8 × 4	Left	Left
24	28	M	hCRH	Right Pit/	14.7	13.4	6.5 × 6.9 × 5	Bilateral	Left
25	28	F	hCRH	Left/Pit	9.5	3.9	9 × 7 × 4	Right	Right
26	22	F	hCRH	Right/Pit	146.8	45.8	6 × 8 × 8	Right	Right
27	25	F	hCRH	Right/Pit	29.5	13.5	8 × 5.3 × 9	Right	Left
28	25	M	hCRH	Right/Pit	20.2	2.1	5 × 3	Right	Right
29	26	F	hCRH	Right/Pit	25.3	18.5	Normal	Normal	Right

### Side effects of drugs used in BIPSS for stimulation and procedure-related complications

Transient hypertension was the commonest side effect associated with use of LVP, while tachycardia was the commonest side effect associated with the use of hCRH. These side effects were mild and transient in nature abated by themselves without any intervention. One patient in the hCRH group developed internal jugular vein thrombosis after the procedure which resolved after anticoagulation therapy. Table 3 shows the profile of side effects observed during the procedure.

**Table 3:** Side effects of lysine vasopressin, hCRH and procedure related.

Side effects	LVP 10 U (*n* = 9)	LVP 5 U (*n* = 10)	hCRH (*n* = 10)
Hypertension	7	6	2
Hypotension	0	0	4
Bradycardia	2	2	0
Tachycardia	0	0	10
Headache	3	4	4
IJV thrombosis	0	0	1
Ear ache	0	0	1
Fall in SpO_2_	1	1	0
Nausea	2	2	0
Pain abdomen	1	1	0
Flushing	0	1	2
Total events	16	17	23

## Discussion

The present study was performed to compare the diagnostic accuracy of lysine vasopressin and hCRH as stimulating agent for the ACTH release during BIPSS to localize and lateralize the source of ACTH in patients with CS. This study showed that both these agents confirmed the source of ACTH excess in all of the patients. BIPSS using 10 units LVP correctly lateralized the pituitary adenoma in more than three-fourth of the patients, which was higher as compared to hCRH or 5 U LVP, although this was not significant statistically. Side effects of both these agents were mild and transient except internal jugular vein thrombosis on right side post procedure in one patient in hCRH group.

Simultaneous BIPSS helps to differentiate between pituitary and the ectopic tumoral ACTH secretion and can provide clue about the site of pituitary adenoma (lateralization). BIPSS is performed in patients of ACTH-dependent CS when there are discordant responses to dynamic testing (intravenous CRH stimulation and HDDST (high-dose dexamethasone suppression test)) or pituitary imaging fails to localize adenoma or reveals pituitary tumor with size ≤6 mm (12, 19, 20, 21, 22). As the ACTH secretion is pulsatile, various drugs have been used to stimulate tumoral corticotropes to improve diagnostic accuracy of BIPSS. Ovine/hCRH, desmopressin or combined hCRH and desmopressin have been used during BIPSS in most of the studies (10, 11, 12, 23, 24). Vasopressin has been shown to be more potent than CRH in stimulating ACTH secretion in animal studies, though it is equipotent to hCRH in human studies when ACTH is measured from the peripheral vein (13). No study has compared the efficacy and potency of vasopressin and hCRH during BIPSS. In our study, we used hCRH or LVP (5 units or 10 units) for ACTH stimulation during BIPSS and compared their efficacy for localizing and lateralizing the ACTH source (16).

In the present study, we found that sensitivity and PPV of BIPSS for localization of ACTH source was 93.1% and 100% at the basal point of time and sensitivity increased to 100% at 3 min after stimulation. Further, in most of the studies, peak central-to-peripheral ratio of 3.0 or more usually occurs between 3 and 5 min post-CRH and desmopressin (7, 26, 27, 28). In a study by Oldfield and coworkers, inferior petrosal sinus: peripheral basal ratio (IPS: P) of ≥2 had sensitivity of 95% and specificity of 100% for localization, while peak IPS: P ratio of ≥3 after ovine CRH administration had the sensitivity and specificity of 100% (7). In one of the largest study by Wind and coworkers, they found that IPSS confirmed a pituitary source of ACTH secretion in 491 patients (98%) and achieved lateralization in 491 (98%) patients (9). A study by Machado and coworkers using desmopressin for stimulation during BIPSS, they found that 47 of the 56 patients achieved a central: peripheral ACTH gradient; 40 patients at baseline (IPS:P ≥2) and seven patients achieved after desmopressin (IPS:P ≥3), achieving a sensitivity of 92.1% and specificity of 100% (23). A study by Tsagarakis and coworkers used combined human CRH and desmopressin for stimulation, and they found that sensitivity and specificity of BIPSS for localization was 97.9% and 100% respectively (24). In a recent study of 6 patients of CS, BIPSS using LVP as stimulus had sensitivity of 80% to localize the source of ACTH production. However, small number of patients, low dose of vasopressin (1 U) and absence of active comparator were the limitations of the study (18).

Further, in most of the studies, BIPSS has been done up to 15–20 min post-stimulation (9, 10, 11) while in some studies it has been performed up to 60 min to 120 min (23, 24, 25). Using LVP for ACTH stimulation during BIPSS, all patients achieved inferior petrosal sinus: peripheral venous peak ACTH ratio of ≥3:1 by 2 min, while using hCRH, it was achieved at 3 min post stimulation in all the patients. Therefore, sampling during BIPSS beyond 5–10 min with either of stimulant is not required.

Maximum stimulated ACTH level of 20,000 pg/mL and 1687 pg/mL were achieved with 5 U and 10 U LVP, respectively, while a maximum stimulated ACTH level of 3848 pg/mL was achieved with 100 µg of hCRH. A study by Wind and coworkers has suggested that peak ACTH level, either basal or stimulated <400 pg/mL without diagnostic IPS:P ratio is suggestive of false-negative result. One of our patient did not achieve this ACTH value of 400 pg/mL but had diagnostic IPS:P ratios (9). The stimulated ACTH response depends on multiple factors like potency of stimulus, tumor size, basal ACTH level and magnitude of expression of vasopressin receptors on the tumor tissue.

Use of simultaneous BIPSS is an extremely powerful technique for establishing the central origin of ACTH secretion; however, its diagnostic accuracy for the lateralization of pituitary microadenomas is limited due to the various factors like inter-individual variability in dominant petrosal sinus drainage, anomalous inter-cavernous sinus venous connections and extension of epicenter of the tumor to the opposite side. Using inter-sinus ratio of ≥1.4:1 for lateralization of corticotrope microadenomas, the diagnostic accuracy of inferior petrosal sinus sampling ranges from 50 to 100%, when compared with findings at pituitary surgery as the ‘gold standard’ (7, 8, 9, 23, 28, 29).

In the present study, accuracy of BIPSS for lateralizing adenoma was 16/22 patients at the basal point of time. Further, after stimulation with hCRH, 5 U LVP and 10 U LVP, accuracy of lateralization observed was 6/10, 7/9 and 7/8 of patients respectively, denominating the much higher though not significant correct lateralization after 10 U LVP stimulation as compared to hCRH or 5 U LVP. This can be explained by the higher potency of 10 U LVP for stimulating the ACTH secretion. In a study by Wind and coworkers, they showed that PPV of lateralization was 69% and left-sided IPSS lateralization with consistent lateralization before and after CRH administration were associated with enhanced accuracy (9). The lateralization depends on multiple factors like potency and specificity of stimulus, tumor size, basal ACTH level and magnitude of expression of vasopressin receptors on tumor tissue.

In our study, concordance rate of CEMRI with BIPSS was 16 out of 24; CEMRI with TSS was 18 out of 24 and BIPSS with TSS was 20 out of 27 patients in lateralizing adenoma, which is comparable to the other studies. In a study by Gillian and coworkers, they found that BIPSS and imaging studies were concordant for 67% of patients and discordant for 33% of patients. When results are discordant, BIPSS predicted the side of lesion better than MRI (63% vs 13%), which is in contrast to our study where CEMRI better correlated with TSS when results are discordant between CEMRI and BIPSS (71.4% vs 28.5%) (30). In another study, Colao and coworkers observed that the side of the adenoma as predicted by BIPSS and magnetic resonance imaging was in agreement with surgical evidence in 65% and 75% of cases, respectively (12). As in most of the studies, our study also demonstrates that the ability of BIPSS in lateralizing adenoma is limited.

Although BIPSS is a safe procedure in the experienced hands but neurological side effects such as medial medullary syndrome, pontine hemorrhage, subarachnoid hemorrhage, groin hematoma or internal jugular venous (IJV) thrombosis have been described (9, 31, 32, 33, 34, 35). In our study, one patient developed IJV thrombosis, which is a known complication of BIPSS, rest of the patients had minor side effects that were transient in nature and did not require specific treatment.

The strengths of the study include correlation of BIPSS results with the intra-operative findings, further confirmation by histopathology and an active comparative group. Moreover, LVP is widely available as compared to CRH, thereby enhancing the utility of BIPSS. Small number of patients is the limitation of the study.

In conclusion, this is the first study comparing the use of LVP and hCRH during BIPSS. Both the stimuli confirmed the source of ACTH excess in all the patients, while 10 U LVP could lateralize the pituitary adenoma in higher number of the patients as compare to hCRH or 5 U LVP.

## Declaration of interest

The authors declare that there is no conflict of interest that could be perceived as prejudicing the impartiality of the research reported.

## Funding

This work did not receive any specific grant from any funding agency in the public, commercial, or not-for-profit sector.
